# The Effectiveness of Anti-*R. equi* Hyperimmune Plasma against *R. equi* Challenge in Thoroughbred Arabian Foals of Mares Vaccinated with *R. equi* Vaccine

**DOI:** 10.1155/2014/480732

**Published:** 2014-04-03

**Authors:** Osman Erganis, Zafer Sayin, Hasan Huseyin Hadimli, Asli Sakmanoglu, Yasemin Pinarkara, Ozgur Ozdemir, Mehmet Maden

**Affiliations:** ^1^Department of Microbiology, Faculty of Veterinary Medicine, University of Selcuk, 42075 Konya, Turkey; ^2^Program of Food Technology, Sarayonu Vocational School, University of Selcuk, 42075 Konya, Turkey; ^3^Department of Pathology, Faculty of Veterinary Medicine, University of Selcuk, 42075 Konya, Turkey; ^4^Department of Internal Medicine, Faculty of Veterinary Medicine, University of Selcuk, 42075 Konya, Turkey

## Abstract

This study aimed to determine the effectiveness of a pregnant mare immunization of a *Rhodococcus equi* (*R. equi*) vaccine candidate containing a water-based nanoparticle mineral oil adjuvanted (Montanide IMS 3012) inactive bacterin and virulence-associated protein A (VapA), as well as the administration of anti-*R. equi* hyperimmune (HI) plasma against *R. equi* challenge in the mares' foals. The efficacy of passive immunizations (colostral passive immunity by mare vaccination and artificial passive immunity by HI plasma administration) was evaluated based on clinical signs, complete blood count, blood gas analysis, serological response (ELISA), interleukin-4 (IL-4) and interferon gamma (IFN-**γ**), total cell count of the bronchoalveolar lavage fluids (BALF) samples, reisolation rate of *R. equi* from BALF samples (CFU/mL), lung samples (CFU/gr), and lesion scores of the organs and tissue according to pathological findings after necropsy in the foals. The vaccination of pregnant mares and HI plasma administration in the foals reduced the severity of *R. equi* pneumonia and lesion scores of the organs and tissue by 3.54-fold compared to the control foals. This study thus indicates that immunization of pregnant mares with *R. equi* vaccine candidate and administration of HI plasma in mares' foals effectively protect foals against *R. equi* challenge.

## 1. Introduction

*Rhodococcus equi *(*R. equi*) is a Gram-positive, nonmotile, obligate aerobe, intracellular microorganism. Virulent* R. equi *causes pyogranulomatous bronchopneumonia in young foals aged from 1 to 6 months [1]. Young foals may also develop extrapulmonary disease, such as septic arthritis, osteomyelitis, ulcerative enterocolitis, mesenteric lymphadenopathy, neonatal diarrhea, and sudden death.* R. equi* is additionally considered as an opportunistic pathogen of immunosuppressed people, especially AIDS patients [2].* R. equi *was initially isolated from pulmonary lesions of foals by Magnusson in 1923 [3].* R. equi* bacterium is present in soil and horse feces. Foals are thought to become infected when, within the first few days of life, they ingest or breathe in soil, dust, or fecal particles harboring the bacteria [2, 4]. Inhalation of aerosolized virulent* R. equi* from the environment and intracellular replication within alveolar macrophages is essential components of pathogenesis of* R. equi* pneumonia in foals [5]. Virulence in foals is associated with the presence of 80–90 kb plasmids that encode the 15–17 kDa lipoprotein “virulence-associated protein A” (VapA) [6]. The disease is endemic on some farms and sporadic on other farms, but nonexistent on most farms. Recent epidemiologic studies indicate that the difference in the disease's prevalence on farms directly relates to differences in foal population density, farm management, and environmental factors, such as temperature, dust, soil pH, and the number of virulent* R. equi *organisms in the soil [7].

Several antimicrobial agents are active against* R. equi *in vitro. However, since* R. equi *is a facultative intracellular pathogen that survives, replicates in macrophages, and causes pyogranulomatous lesions, many of these agents are ineffective in vivo [8].* R. equi *pneumonia significantly impacts the equine industry by posing financial losses since foals that recover from the disease are less likely to race as adults. The cost of therapy and occasional death of foals also pose financial risks. Furthermore, treatment with long-term antibiotics does not guarantee full recovery [9].

Due to the epitheliochorial placentation of equines, foals must obtain all maternally derived antibodies from the ingestion of colostrum [10]. Ingestion of colostrum from hyperimmunized mares was found to be associated with protection against* R. equi* in foals normally hypogammaglobulinemic at birth [11, 12]. Foals become infected approximately when maternal antibody concentrations wane [13]. Immunization of mares has been suggested by several researchers to prevent* R. equi* infection in foals [11, 12, 14–17]. Traditional hyperimmune plasma therapy is currently the only proven method for prevention of* R. equi* in foals, especially those exhibiting passive antibody transfer failure [11, 12, 15].

Due to the presence of the maternal antibody and the immaturity of foals' immune system, vaccination of neonate presents different results [18–20], yet none of the control strategies to protect horses from* R. equi* infection have proven successful. Several vaccines have been investigated for the prevention of* R. equi*, though none have been developed for widespread vaccination [21].

This study thus aimed to determine the effectiveness of a pregnant mare immunization with a* R. equi* vaccine candidate and the administration of anti-*R. equi* hyperimmune plasma against* R. equi* challenge in these mares' foals.

## 2. Materials and Methods

### 2.1. Immunization of Mares

Four pregnant thoroughbred Arabian mares were vaccinated three times at months 8, 9, and 10 during pregnancy. Vaccination was performed intramuscularly with the* R. equi* vaccine candidate containing a water-based nanoparticle mineral oil adjuvanted (IMS 3012, SEPPIC, Paris, France) inactive antigen and VapA. Four mares not vaccinated formed the control group. Serum samples were collected from each mare at birth to test the presence of an anti-*R. equi*-specific antibody by ELISA. ELISA was carried out according to Takai et al. [22].

Nine healthy Arabian mares were selected for the production of anti-*R. equi* hyperimmune plasma. After proving to be free of equine infectious anemia (EIA), dourine, glanders, African horse sickness, and* S. abortus equi*, the mares were hyperimmunized 5 days apart with four doses of inactive* R. equi*. After 10 to 15 days, mares were vaccinated 21 days apart with 3 doses of* R. equi* vaccine candidate [23, 24]. After 15 to 20 days following the most previous immunization, serum samples were obtained from the mares and tested by ELISA for anti-*R. equi* antibody titers [22]. Horses having anti-*R. equi* antibody titers ≥1/12800 by ELISA were selected as plasma donors. Donor horses were bled, and the hyperimmune plasma was separated from the blood cells by plasmapheresis (PCS2, Haemonetics, Braintree, MA, USA). The plasma samples were packed in 200 mL sterile bottles in a BSL 2 cabinet and stored at 4°C. Sterility tests for aerobic, anaerobic bacteria, mycoplasma, and mycotic agents as well as mouse safety tests were performed, after which the hyperimmune plasma samples were used. Donor horses were subsequently vaccinated at intervals of 50 to 60 days and tested 10 to 15 days later, and if the titers were again satisfactory, they were again bled.

### 2.2. Challenge

To determine the effectiveness of a pregnant mare immunization using a* R. equi* vaccine candidate and HI plasma activity against* R. equi* infection in foals, 4 weeks old mares which born four vaccinated and four unvaccinated mares challenged the 2 mL of 1.0 × 10^5^ CFU pathogen* R. equi* by intercostal injection in the lobe of the left lung [25, 26]. Before receiving the challenge, foals were kept together with their dams approximately 3 weeks after birth to ingest a sufficient amount of colostrum. Two days before the challenge, 150 mL of HI plasma was administered to each foal of the vaccinated mares by intravenous infusion and 50 mL by subcutaneous infusion at days 1, 5, 9, 13, and 17 after the challenge. HI plasma was not given to the foals of unvaccinated mares.

### 2.3. Laboratory Tests

Blood samples were obtained from the challenged foals to determine the presence of anti-*R. equi* specific antibodies using a ELISA on the challenge day (day 0) and on days 10 and 20, to measure the interleukin-4 (IL-4) and interferon gamma (IFN-*γ*) concentrations on days 0, 1, 10, 20, and 30 and for complete blood count and blood gas analysis on days 0, 1, 5, 10, 14, 20, and 30 [25]. BALF samples were taken by passing a nasotracheal tube and in fusing 20 mL of sterile saline solution to bacterial culture and measure total cell count (TCC) on days 0, 1, 5, 10, 14, 20, and 30 [25, 26]. BALF samples were collected according to the method described by Mansmann and Knight [27] and Higuchi et al. [28]. The IL-4 and IFN-*γ* levels were measured using the Horse IL-4 ELISA kit (product code CSB-E14223Hs [96 T], Cusabio, Wuhan, Hubei, China) and Equine IFN-*γ* ELISA kit (ALP) (product code 3117-1A-6, MabTech, Thomastown, VIC, Australia) according to the manufacturer's instructions. The optimal dilutions of the serum samples, conjugate, substrate, and concentration of the coating antigen were standardized in our laboratory.

### 2.4. Clinical Examination

Fever (F), respiratory rate (R), pulse rate (P), cough, bronchial sounds, nasal discharge, and mucous membranes of foals were examined on days 0, 1, 5, 10, 14, 20, and 30. Clinical and respiratory system signs were defined as regular (0), mild (1), moderate (2), and severe (3) and normal (0), congested (1), and cyanotic (2) for mucous membranes.

### 2.5. Postmortem Examination

Foals were euthanized with an intravenous injection of a mixture of 100 mg of suxamethonium chloride and 22.5 mg sodium chloride (Lysthenon 2%, Fako, Turkey) 30 days after the challenge, and necropsy was performed. Macroscopic lesions in lungs and organs were scored according to Table 1.

Samples from these organs were fixed in 10% formalin and embedded in paraffin wax blocks. Block sections of 5 µm thickness were stained with Luna [29]. All sections were evaluated under a light microscope.

### 2.6. Statistical Analysis

All statistical analyses were performed using the Student's *t*-test.

## 3. Results

Two foals (numbers 5 and 7) of unvaccinated mares died before the challenge, and* R. equi*,* Streptococcus* sp., and* Corynebacterium* sp. were isolated from the lungs of these dead foals. In the place of the dead foals, two unvaccinated mares' foals were included in the study. One of these foals died on day 7 and another on day 13 after the challenge.

Anti-*R. equi* antibody titer measured in vaccinated mares was higher than in the unvaccinated mares at birth by ELISA. In the foals of vaccinated mares, anti-*R. equi *antibody titer was determined to be 1/1600 of the maximum after the administration of hyperimmune plasma, and 1/200 titer was also determined in control foals (Table 2).

The IL-4 concentration was measured to have a mean of <16 pg/mL at days 0, 10, and 20 and a mean of 80 pg/mL at day 30 in foals of the vaccinated group, as well as a mean of 32 pg/mL in control foals. IFN-*γ* concentration increased on day 10 compared to the challenge day in both vaccine and control groups. By day 20, the control group remained stable, while the vaccine group exhibited doubling. By day 30, it increased in both groups but was measured to be 4.1-fold more in the vaccine group than in the control group (Table 3).

**Figure 1:**
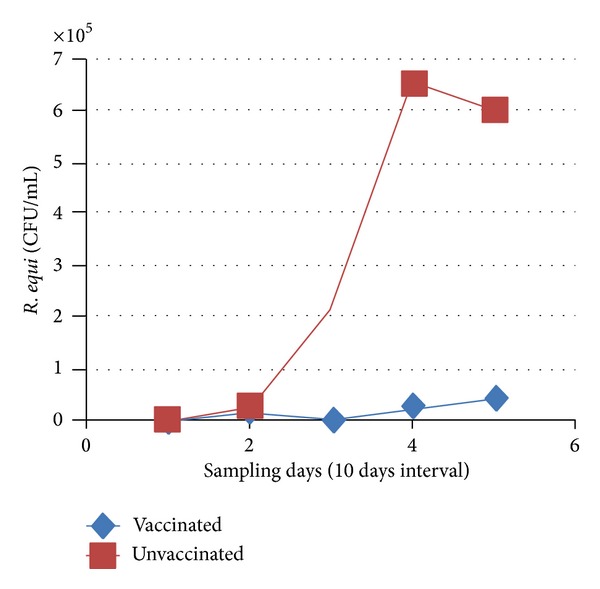
Reisolation rate of* R. equi* from foals' BALF samples (CFU/mL).

The reisolation rate of* R. equi *from BALF samples was determined to have increased by day 10, decreased by day 20, and increased again by day 30 in the vaccinated and HI plasma-administered group. An increase was observed in the control group compared to day 1 (Table 4, Figure 1).

A decrease in the concentration of pO2 and SO2 in the vaccine group was observed on day 5. The concentration of pCO2 and tCO2 increased, while the concentration of SO2 decreased on day 10. A decrease in the concentration of pO2 and SO2 in the control group was observed on day 30 (*P* < 0.05) (Table 6).

An insignificant (*P* > 0.05) increase was observed in WBC concentration in both the control and vaccine group during the study period. Significant (*P* < 0.05) differences were determined in percentages of LYM, MON, and GRAN concentration. The RBC concentration decreased significantly (*P* < 0.05) on days 14 and 30 (Table 6).

TCC of BALF and lung scores had increased in both groups by day 14 (Table 5). These increases were statistically significant (*P* < 0.05) in the control group, yet insignificant (*P* > 0.05) in the vaccine group.

The clinical and lung auscultation findings indicate that clinical signs commenced on day 5 and increased until day 10 in both groups. In the vaccine group, however, it began to decrease after day 10 and because of the sepsis two foals died on days 7 and 15 in the control group.

**Figure 2:**
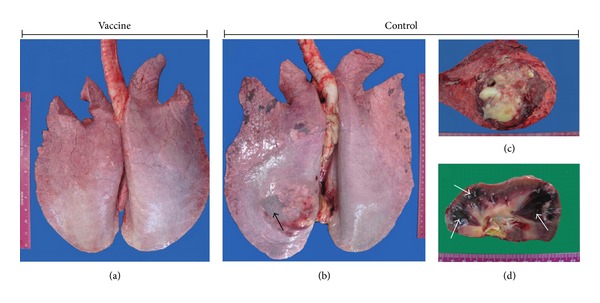
(a) Normal lung in a foal of vaccine group, (b) pyogranuloma formation in the* R. equi* inoculated left lung of a control foal (arrow), (c) pyogranuloma in lung, and (d) infarction areas in kidney of control foals (arrows).

The mean of total lesion scores of the organs and tissue was determined to be 78 in control group and 22 in the vaccine group. According to pathological findings, the severity of* R. equi* pneumonia and lesion scores of the organs and tissue was observed 3.54-fold less in the vaccinated and HI plasma-administered foals compared to the control foals (Table 7, Figure 2).

## 4. Discussion

Cell-mediated immunity is thought to play an important role in eliminating the facultative intracellular pathogen from foals, yet humoral immunity seems to be critically involved in the early protection in young foals. Foals are the most susceptible to the effects of virulent organisms when maternal antibody levels wane [30, 31]. The passive transfer of immunity plays a critical role in the foals' resistance to a variety of infectious agents. Due to the epitheliochorial placentation of equines, foals must obtain all of their maternally derived antibodies by ingesting colostrum [10]. The lowest circulating antibody titers in foals appear from 1 to 6 months via the combined effects of waning maternally derived antibodies and low endogenous antibody production [32]. As a result, foals are susceptible to* R. equi* pneumonia during this period. Due to age-dependent susceptibility to* R. equi*, foals need to develop anti-*R. equi *immunity shortly after birth [25].

Since* R. equi* lives within macrophages, it resists many common antibiotics, and antibiotics-based therapy is prolonged, expensive, possibly associated with adverse effects, and inconsistently successful [33].

Studies investigating the active immunization of mares as a means of enhancing the passive transfer of virulent* R. equi *antibodies in colostrum and protecting foals from* R. equi* pneumonia have yielded mixed results. Solo vaccination of mares has not proven protective against* R. equi *pneumonia in foals, despite a significant increase in a colostral-specific antibody [14, 34]. Martens et al. [34], Madigan et al. [35], and Varga et al. [36] did not observe protection in foals against* R. equi *pneumonia after mare vaccination. Moreover, Hines et al. [31] reported that immunoglobulin in mares may not be efficiently transferred via colostrum. However, according to other researchers, passive antibody transfer from ingested colostrum was found to be associated with protection against* R. equi* in foals normally hypogammaglobulinemic at birth [11, 12, 14, 15]. Immunization of pregnant mares with virulent* R. equi* and VapA protein antigen associated with a water-based nanoparticle adjuvant as a candidate vaccine developed a higher serum IgG and opsonic activity, which resulted in passive antibody-mediated protection of foals [14]. Muscatello [17] reported that the protective effect was associated with an increase in the opsonic capacity of polymorphonuclear leucocytes against virulent* R. equi* in foals from immunized mares.

Martens et al. [37] were the first to show the immunoprophylactic capacity of specific hyperimmune plasma in an experimental model of* R. equi* pneumonia in foals. Other researchers have reported a reduction in foal morbidity and mortality due to* R. equi* as a result of HI plasma administration [14, 35, 37–39]. However, several other studies report no protective effects of HI plasma [11, 12, 40].

According to our results, the immunization of pregnant mares with a* R. equi* vaccine candidate and the administration of the anti-*R. equi* HI plasma in vaccinated mares' foals proved to have protective effects during experimental* R. equi* infection. The clinical signs of pneumonia were significantly delayed, and the reisolation rate of* R. equi *from BALF samples decreased. The natural mortality rate due to* R. equi* infection was less than 50% in these foals compared to the controls. The severity of* R. equi* pneumonia and lesion scores of the organs and tissue determined 3.54-fold less than control foals.

The protective components of HI plasma are not completely known. Antibodies to Vap proteins, specifically VapA, appear to be crucially important [17]. It has been reported that there is no correlation between the total serum IgG levels and concentration of the specific anti-*R. equi* antibody [34, 35]. The phagocytic ability of foal neutrophils has been found comparable to adults, but the lymphocyte stimulation response alone did not influence the course of* R. equi* infection, while the opsonic ability of foal serum was found to be a limited factor for phagocytosis from the ages of 1 to 6 weeks [41, 42]. Phagocytic activity of foal neutrophils was found to improve when mixed with adult serum or plasma [43], which may be related to unknown, nonspecific immune factors provided by HI plasma and normal adult equine plasma that are absent from colostrum, such as fibronectin, complement, and cytokines [12, 40, 44]. The effectiveness of HI plasma is likely to be affected by the dosage, timing of administration, innate immune system competence, management conditions, and number of virulent bacterin in the environment [45].

Cytokines IFN-*γ* and IL-4 are major macrophage and neutrophil-activating factors, as well as upregulated microbial killing pathways [46]. It is reported that newborn foals had a deficiency of IFN-*γ*/IL-4 and levels not reaching adult status until approximately 4 months of age [47]. Reduced IFN-*γ* and IL-4 expression have a limited killing capacity of phagocytes in young foals [47, 48]. In our study, the IFN-*γ* and IL-4 concentration measured 4.1-fold and 2.5-fold more in the foals of vaccinated mares and HI plasma-administered, respectively, compared to the control foals.

According to changes in blood gases of foals, it was observed that lung ventilation in the challenge group had been affected by day 5, intensified after day 10, and continued to increase during the study period.

The important decrease (*P* < 0.05) of the RBC concentration on days 14 and 30 and significant differences (*P* < 0.05) of the MCV and RDW values in the vaccine and control group were evaluated as microcytic/normocytic-normochromic-regenerative anemia which was interpreted as a response to emerging infectious.

Given the decrease in the number in the controls, BALF-TCC parameters were detected as statistically insignificant (*P* > 0.05).

According to clinical scores and laboratory findings, the effect of infection in the vaccine group began on day 5, increased from day 9 to 14, and was constant from days 20 to 30. The effect of infection was similar in the control group, though the clinical findings increased from days 7 to 15, and two foals died of sepsis during this latter period. These findings show that resistance to infection was low in the control group.

As yet there is no licensed vaccine for the prevention of* R. equi*. However, on January 27, 2011, Intervet/Schering-Plough Animal Health announced that a vaccine against* Rhodococcus equi* infection in foals had entered the final stages of development [49].

## 5. Conclusions

Our results indicate that the immunization of pregnant mares with a water-based nanoparticle mineral oil adjuvanted (IMS 3012) inactive bacterin and VapA and the administration of HI plasma in foals of these mares effectively protect foals against* R. equi *challenge. Foals are born into the* R. equi* contaminated environment due to mares carrying the* R. equi* in their intestines.* R. equi* infection can be controlled by both the mares' vaccination and anti-*R. equi*-HI plasma administration in foals of such dams.

## Figures and Tables

**Table 1:** Macroscopic lesions scores in lungs and organs after necropsy.

Postmortem Lesion Scores					
Organs	No lesion (0)	Mild (1)	Moderate (2)	Severe (3)	Very severe (4)
Lung	Without lesions	With 2 to 3 small pyogranulomas or large abscess formation limited to only one lobe of the lung and focal consolidation	With four to five pyogranulomas limited to two lobes of the lung multifocal consolidation and large abscess formation	With a large number of differently sized pyogranulomas limited to three lobes or between the right and left lobes of the lung	With military or differently sized pyogranulomas spread over all lobes of the lung

Lymph node	Without lesions	With slight growth	With mild growth	Overgrown and with limited pus foci of the cross-sectional surface	Overgrown and purulent

Heart	Without lesions	With slight paleness	With a wide area of paleness in the epicardium and endocardium or pyogranuloma	With a wide area of paleness in the cross-sectional area or a few pyogranulomas	With a wide area of paleness in the cross-sectional area or several pyogranulomas

Liver	Without lesions	Slightly enlarged and congested	Enlarged and mottled appearance or with pyogranuloma	With numerous pyogranulomas in the cross-sectional area and under the capsule	With pyogranulomas spread over the entire surface and the cross-sectional area

Kidney	Without lesions	Slightly enlarged	Enlarged kidneys and mottled appearance	Enlarged kidneys and with pyogranuloma in the cross-sectional area and infarction area	Enlarged kidneys and with numerous pyogranulomas in the cross-sectional area and infarction area

Spleen	Without lesions	Slightly enlarged	Overgrown and with pyogranuloma in the cross-sectional area	Overgrown and with a few pyogranulomas in the cross-sectional area	Overgrown and with numerous pyogranulomas in the cross-sectional area

**Table 2:** Anti-*R. equi* antibody ELISA titer of vaccinated and unvaccinated mares and challenged foals.

Mare group	Mare number	ELISA titer at birth	Foal group	Foal number	ELISA titer after challenge		
					Day 0	Day 10	Day 20
Vaccinated	1	1/12800	Vaccine + HI plasma	1	1/800	1/1600	1/1600
	2	1/6400		2	1/100	1/400	1/800
	3	1/3200		3	1/800	1/1600	1/1600
	4	1/6400		4	1/400	1/800	1/800

Unvaccinated	5	Negative	Control	5	0	1/200	∗
	6	1/200		6	0	Negative	1/200
	7	Negative		7	0	∗	∗
	8	Negative		8	0	Negative	1/200

*Because foals died, ELISA titer was not measured on these days.

**Table 3:** IL-4 (pg/mL) and IFN-*γ* (ng/mL) concentration in challenged foals.

Foal group	Foal number	Day 0	Day 10	Day 20	Day 30
		IL-4/IFN-*γ*	IL-4/IFN-*γ*	IL-4/IFN-*γ*	IL-4/IFN-*γ*
Vaccine + HI plasma	1	<16/<0.01	<16/0.01	<16/0.02	**128/0.05**
	2	<16/<0.005	<16/0.005	<16/0.01	**128/0.05**
	3	<16/<0.005	<16/0.005	<16/0.01	32/0.05
	4	<16/<0.005	<16/0.005	<16/0.01	32/0.05
	Mean	**<16/<0.0075**	**<16/0.0075**	**<16/0.015**	**80/0.05**

Control	5	<16/<0.005	<16/0.005	∗	∗
	6	<16/<0.005	<16/0.005	<16/0.001	32/0.005
	7	<16/<0.005	∗	∗	∗
	8	<16/<0.005	<16/0.005	<16/0.01	32/0.02
	Mean	**<16/<0.005**	**<16/0.005**	**<16/0.0055**	**32/0.012**

*Because the foals died, IL-4 and IFN-*γ* concentration were not measured on these days.

**Table 4:** Reisolation rate of *R. equi* from BALF samples (CFU/mL) and lung samples (CFU/gr) after necropsy in foals.

Foal group	Foal number	Day 0	Day 1	Day 10	Day 20	Day 30	Necropsy
Vaccine + HI plasma	1	0	65000	135	1400	140	50
	2	0	1000	55	1220	0	21000
	3	0	1300	120	12700	0	0
	4	0	2600	195	90000	170000	∗∗
	Mean	**0**	**17475**	**126**	**26330**	**42535**	

Control	5	0	19000	800	died	—	∗∗
	6	0	4100	3600	1300000	1100000	320000
	7	0	5500	Died	—	—	∗∗
	8	0	83000	640000	2300	100000	3250000
	Mean	**0**	**27900**	**214666**	**651150**	**600000**	

**Too many to count.

**Table 5:** Total cell count (TCC) of BALF samples, clinical signs, respiratory system signs, mucous membrane scores, and temperature of foals.

	Foal group	Day 0	Day 1	Day 5	Day 10	Day 14	Day 20	Day 30
BALF-TCC	Control	300.00 ± 57.74	300.00 ± 57.74	300.00 ± 57.74	300.00 ± 57.74	466.67 ± 266.67	8800.00 ± 8400.00	1500.00 ± 1100.00
	Vaccinated	**300.00 ± 57.74**	**300.00 ± 57.74**	**300.00 ± 57.74**	**300.00 ± 57.74**	**2600.00 ± 1116.54**	**1000.00 ± 258.20**	**1950.00 ± 1040.43**

Clinical score	Control	0.00 ± 0.00	0.00 ± 0.00	0.00 ± 0.00	0.00 ± 0.00	1.00 ± 1.00	0.00 ± 0.00	0.00 ± 0.00
	Vaccinated	**0.00 ± 0.00**	**0.00 ± 0.00**	**0.00 ± 0.00**	**0.00 ± 0.00**	**0.00 ± 0.00**	**0.00 ± 0.00**	**0.00 ± 0.00**

Respiratory system score	Control	0.00 ± 0.00	0.00 ± 0.00	**0.00 **± 0.00 B	1.00 ± 0.58	0.67 ± 0.33 B	0.00 ± 0.00 B	0.00 ± 0.00 B
	Vaccinated	**1.00 ± 0.00**	**0.00 ± 0.00**	**1.25 ± 0.48 A**	**2.00 ± 0.00**	**0.275 ± 0.25 A**	**2.75 ± 0.25 A**	**2.75 ± 0.25 A**

Mucous membrane score	Control	0.00 ± 0.00	0.00 ± 0.00	0.00 ± 0.00	0.00 ± 0.00	0.33 ± 0.33	0.00 ± 0.00	0.00 ± 0.00
	Vaccinated	**0.00 ± 0.00**	**0.00 ± 0.00**	**0.00 ± 0.00**	**0.00 ± 0.00**	**0.00 ± 0.00**	**0.00 ± 0.00**	**0.00 ± 0.00**

Temperature	Control	38.3 ± 0.12	38.43 ± 0.11	38.05 ± 0.23	27.90 ± 9.31	37.87 ± 0.24	38.20 ± 0.20	38.45 ± 0.05
	Vaccinated	**38.13 ± 0.13**	**38.33 ± 0.22**	**38.50 ± 0.11**	**38.45 ± 0.06**	**38.05 ± 0.16**	**38.60 ± 0.07**	**38.60 ± 0.07**

**Table 6:** Complete blood count and blood gas analysis results of foals.

Foal group		Day 0	Day 1	Day 5	Day 10	Day 14	Day 20	Day 30
pH (7.34–7.43)	Control	7.45 ± 0.02	7.41 ± 0.02	7.43 ± 0.02	7.35 ± 0.03	7.42 ± 0.01	7.45 ± 0.02	7.40 ± 0.02
	Vaccinated	**7.43 ± 0.01**	**7.42 ± 0.01**	**7.41 ± 0.01**	**7.41 ± 0.01**	**7.41 ± 0.01**	**7.39 ± 0.01**	**7.42 ± 0.01**

pCO2 (38–48 mmHg)	Control	37.50 ± 1.19	42.25 ± 0.75	45.75 ± 1.93	52.75 ± 2.78 A	47.33 ± 2.73	40.50 ± 1.50	47.00 ± 3.00
	Vaccinated	**40.00 ± 1.78**	**42.00 ± 1.47**	**42.00 ± 0.41**	**40.25 ± 125 B**	**42.00 ± 2.74**	**45.00 ± 3.14**	**40.50 ± 1.26**

pO2 (37–56 mmHg)	Control	35.00 ± 2.12	35.00 ± 2.12	36.75 ± 1.55 A	33.00 ± 1.35	32.00 ± 2.08	38.00 ± 4.00	34.00 ± 1.01 B
	Vaccinated	**37.25 ± 3.71**	**33.50 ± 2.63**	**29.00 ± 1.91 B**	**36.25 ± 1.75**	**53.75 ± 21.46**	**33.75 ± 2.29**	**41.25 ± 1.38A**

tCO2	Control	26.98 ± 1.26	28.03 ± 1.21	31.53 ± 1.81	30.53 ± 0.61 A	31.87 ± 1.18	29.05 ± 0.55	30.20 ± 0.90
	Vaccinated	**27.85 ± 0.45**	**28.35 ± 0.73**	**28.05 ± 0.29**	**26.75 ± 0.84 B**	**27.95 ± 1.47**	**54.15 ± 27.30**	**27.65 ± 0.57**

SO2 (>60 mmHg)	Control	70.00 ± 2.48	67.00 ± 3.37	71.50 ± 1.32 A	71.50 ± 1.32 A	62.67 ± 4.67	74.50 ± 4.50	65.00 ± 1.00 B
	Vaccinated	**71.25 ± 6.70**	**64.75 ± 4.87**	**55.50 ± 4.29 B**	**55.50 ± 4.29 B**	**71.50 ± 9.50**	**53.90 ± 7.42**	**77.75 ± 1.38 A**

WBC (5–12 × 10^3^)	Control	7.08 ± 1.01	7.68 ± 1.31	7.32 ± 1.16	7.02 ± 1.12	5.65 ± 1.66	4.64 ± 0.37	10.53 ± 2.61
	Vaccinated	**6.66 ± 0.87**	**5.86 ± 0.33**	**8.82 ± 1.29**	**8.86 ± 0.87**	**8.60 ± 0.66**	**26.90 ± 15.77**	**10.33 ± 1.57**

LYM (% 20–40)	Control	15.60 ± 2.46 B	15.70 ± 0.90 B	22.65 ± 2.41	22.80 ± 1.86 B	29.13 ± 11.52	36.85 ± 14.75	17.50 ± 10.00
	Vaccinated	**35.60 ± 3.98 A**	**30.65 ± 2.23 A**	**34.63 ± 6.43**	**36.75 ± 4.88 A**	**37.83 ± 4.26**	**27.11 ± 6.51**	**31.85 ± 3.40**

MON (% 2–8)	Control	1.40 ± 0.12 B	1.68 ± 0.11	3.55 ± 0.13	3.43 ± 0.77	3.67 ± 1.22	3.50 ± 0.90	1.60 ± 0.70 A
	Vaccinated	**2.75 ± 0.45 A**	**2.73 ± 0.52**	**3.00 ± 0.48**	**3.05 ± 0.46**	**3.58 ± 0.33**	**8.00 ± 5.90**	**2.53 ± 0.46**

GRAN (% 50–70)	Control	83.00 ± 2.55 A	82.63 ± 0.92 A	73.80 ± 2.46	73.78 ± 2.32	67.20 ± 12.72	59.65 ± 15.65	80.90 ± 10.70
	Vaccinated	**61.65 ± 3.70 B**	**64.75 ± 4.87 B**	**62.38 ± 6.69**	**60.20 ± 5.10**	**58.60 ± 4.42**	**49.43 ± 16.35**	**65.63 ± 3.78**

RBC (7–13 × 10^6^)	Control	8.87 ± 0.48	9.28 ± 0.89	9.27 ± 0.39	9.10 ± 0.71	8.10 ± 0.64 B	9.01 ± 1.16	8.05 ± 0.24 B
	Vaccinated	**9.65 ± 0.32**	**11.30 ± 1.02**	**10.22 ± 0.34**	**10.03 ± 0.22**	**9.89 ± 0.26 A**	**25.16 ± 15.82**	**9.98 ± 0.27 A**

MCV (35–60 fl)	Control	32.63 ± 0.29 A	32.83 ± 0.24 A	32.78 ± 0.71 A	34.48 ± 0.47 A	33.23 ± 0.91	32.70 ± 0.90	32.00 ± 1.90
	Vaccinated	**29.43 ± 1.14 B**	**29.95 ± 0.76 B**	**29.50 ± 0.93 B**	**29.30 ± 0.99 B**	**30.85 ± 0.92**	**26.24 ± 5.43**	**30.50 ± 0.68**

HCT (% 32–53)	Control	28.85 ± 1.39	30.43 ± 3.04	30.30 ± 1.10	31.38 ± 2.66	26.77 ± 1.39	29.30 ± 3.00	25.65 ± 0.75 B
	Vaccinated	**28.43 ± 1.98**	**33.70 ± 2.90**	**30.10 ± 1.11**	**29.35 ± 1.42**	**30.43 ± 0.92**	**29.18 ± 0.71**	**30.33 ± 0.50 A**

MCHC (30–42 g/dL)	Control	39.75 ± 0.43	36.18 ± 2.50	39.63 ± 0.26	36.78 ± 0.86	37.43 ± 0.45	38.85 ± 0.15	40.50 ± 1.20
	Vaccinated	**39.50 ± 1.67**	**40.60 ± 0.94**	**43.65 ± 3.07**	**41.73 ± 1.15**	**37.93 ± 0.61**	**38.40 ± 0.93**	**38.70 ± 1.09**

RDW (8–12)	Control	14.45 ± 0.10	14.60 ± 0.47 B	14.30 ± 0.27 B	14.10 ± 0.43 B	14.13 ± 0.29	15.05 ± 0.85	16.65 ± 0.65
	Vaccinated	**15.40 ± 0.54**	**16.00 ± 0.31 A**	**15.85 ± 0.37 A**	**16.18 ± 0.55 A**	**16.30 ± 0.83**	**16.70 ± 1.00**	**17.15 ± 0.84**

HB (11–17 g/dL)	Control	11.48 ± 0.47	10.83 ± 0.59	12.03 ± 0.51	11.53 ± 0.86	10.03 ± 0.64	11.40 ± 1.20	10.40 ± 0.00
	Vaccinated	**11.18 ± 0.55**	**13.68 ± 1.12**	**12.40 ± 0.44**	**12.23 ± 0.43**	**11.55 ± 0.39**	**11.23 ± 0.44**	**11.75 ± 0.48**

THR (100–400 m/mm^3^)	Control	278.50 ± 87.36	330.50 ± 87.36	287.00 ± 44.53	238.50 ± 29.83 B	171.33 ± 30.69	240.00 ± 37.00	241.50 ± 8.50
	Vaccinated	**248.50 ± 30.01**	**317.00 ± 40.26**	**410.00 ± 49.83**	**384.50 ± 9.56 A**	**244.00 ± 19.20**	**341.50 ± 26.38**	**279.00 ± 25.65**

**Table 7:** Lesion scores of the organs and tissue according to pathological findings.

Foal group	Foals number	Lung	Bronchial LN	Mediastinal LN	Heart	Liver	Kidney	Spleen	Cecum LN	Total foal score	Total group score	Mean group score
Vaccine + HI plasma	1	1	1	0	0	0	0	0	2	4	** 22**	** 5.5**
	2	0	2	2	0	0	0	1	1	6		
	3	1	1	1	0	0	0	0	0	3		
	4	3	3	2	0	0	0	0	1	9		

Control	5	4	4	4	4	4	4	4	4	32	** 78**	** 19.5**
	6	3	1	1	2	0	0	0	1	8		
	7	4	4	4	4	4	4	4	4	32		
	8	3	1	1	0	0	0	0	1	6		
